# Multivariate Information Fusion With Fast Kernel Learning to Kernel Ridge Regression in Predicting LncRNA-Protein Interactions

**DOI:** 10.3389/fgene.2018.00716

**Published:** 2019-01-15

**Authors:** Cong Shen, Yijie Ding, Jijun Tang, Fei Guo

**Affiliations:** ^1^School of Computer Science and Technology, College of Intelligence and Computing, Tianjin University, Tianjin, China; ^2^School of Electronic and Information Engineering, Suzhou University of Science and Technology, Suzhou, China; ^3^Department of Computer Science and Engineering, University of South Carolina, Columbia, SC, United States

**Keywords:** lncRNA-protein interactions, multiple kernel learning, fast kernel learning, kernel ridge regression, gene ontology

## Abstract

Long non-coding RNAs (lncRNAs) constitute a large class of transcribed RNA molecules. They have a characteristic length of more than 200 nucleotides which do not encode proteins. They play an important role in regulating gene expression by interacting with the homologous RNA-binding proteins. Due to the laborious and time-consuming nature of wet experimental methods, more researchers should pay great attention to computational approaches for the prediction of lncRNA-protein interaction (LPI). An in-depth literature review in the state-of-the-art *in silico* investigations, leads to the conclusion that there is still room for improving the accuracy and velocity. This paper propose a novel method for identifying LPI by employing Kernel Ridge Regression, based on Fast Kernel Learning (LPI-FKLKRR). This approach, uses four distinct similarity measures for lncRNA and protein space, respectively. It is remarkable, that we extract Gene Ontology (GO) with proteins, in order to improve the quality of information in protein space. The process of heterogeneous kernels integration, applies Fast Kernel Learning (FastKL) to deal with weight optimization. The extrapolation model is obtained by gaining the ultimate prediction associations, after using Kernel Ridge Regression (KRR). Experimental outcomes show that the ability of modeling with LPI-FKLKRR has extraordinary performance compared with LPI prediction schemes. On benchmark dataset, it has been observed that the best Area Under Precision Recall Curve (AUPR) of 0.6950 is obtained by our proposed model LPI-FKLKRR, which outperforms the integrated LPLNP (AUPR: 0.4584), RWR (AUPR: 0.2827), CF (AUPR: 0.2357), LPIHN (AUPR: 0.2299), and LPBNI (AUPR: 0.3302). Also, combined with the experimental results of a case study on a novel dataset, it is anticipated that LPI-FKLKRR will be a useful tool for LPI prediction.

## 1. Introduction

Long non-coding RNAs (lncRNAs) constitute a large class of transcribed molecules. They have a characteristic length of more than 200 nucleotides which do not encode proteins (St Laurent et al., 2015). Existing research has proven that lncRNAs can control gene expression during the transcriptional, post-transcriptional, and epigenetic procedures through interacting with the homologous RNA-binding proteins (Guttman and Rinn, 2012; Quan et al., 2015; Tee et al., 2015). A most recent research found that, a kind of lncRNA named lnc-Lsm3b can refrain the activity of the receptor RIG-I, by the induction of viruses during the regulation of immune response (Jiang et al., 2018). This is consistent with previous studies which have proven that lncRNAs are playing potential roles in complex human diseases (Li et al., 2013). Due to the laborious and time-consuming nature of wet experimental methods in molecular biology, many state-of-the-art computational researches have been carried out dealing with the conundrum, in an effort to enhance accuracy and time efficiency (Zou et al., 2012; Jalali et al., 2015; Han et al., 2018).

Since it is very difficult to extract any actual details on the 3D structures of lncRNAs and relative proteins, many sequence-based and secondary structure-based approaches for the prediction of lncRNA-protein interaction (LPI) have been published in the literature. Bellucci et al. have established the well-known catRAPID (Bellucci et al., 2011) by leveraging both physicochemical properties and secondary structure information, which could be employed as compound information to handle the problem of predicting LPI. Meanwhile, the hybrid schema RPISeq has been introduced by Muppirala et al. (2011), which employs both Support Vector Machines (SVM) and Random Forest (RF). Wang et al. have proposed a classifier combining Naive Bayes (NB) and Extended NB (ENB) classifier to extrapolate LPI (Wang et al., 2012). Lu et al. have established lncPro, which translates each LPI into numerical form, and applies matrix multiplication (Lu et al., 2013). Suresh et al. developed RPI-Pred based on SVM, by using the structure and sequence information of lncRNAs and proteins (Suresh et al., 2015).

In contrast to the aforementioned works, Li et al. have introduced the LPIHN by employing an heterogeneous network, assembled with a kind of random walk on lncRNA-protein association profile, with a restart mechanism (RWR) (Li et al., 2015). Ge et al. have used resource allocation mode on a dichotomous network, and they have published the algorithm as LPBNI (Ge et al., 2016). Lately, Hu et al. have proposed a kind of semi-supervised link prediction scheme, entitled LPI-ETSLP (Hu et al., 2017), which was soon upgraded to the IRWNRLPI. This method actually integrates RWR and matrix factorization (Zhao et al., 2018).

Zhang et al. have suggested two classes of state-of-the-art computational intelligence approaches (Zhang et al., 2017). The first includes supervised LPI binary classifiers, which do not require prior knowledge of interactions as negative instances (Bellucci et al., 2011; Muppirala et al., 2011; Wang et al., 2012; Lu et al., 2013; Suresh et al., 2015). second category includes semi-supervised approaches which combine known interactions to suggest unknown LPI. The following are characteristic cases of this class: LPIHN (Li et al., 2015), LPBNI (Ge et al., 2016), LPI-ETSLP (Hu et al., 2017), and IRWNRLPI (Zhao et al., 2018).

Transfer learning (Jonathan et al., 1995), which can recognize and leverage skills or knowledge learned in previous tasks to novel tasks, is viewed as a kind of burgeoning machine learning branch. Whereas, zero-shot learning in pairwise learning with two-step Kernel Ridge Regression (KRR) (Stock et al., 2016), is a special type of transfer learning, constructing predictors from a dataset which contains both labeled and unlabeled samples. Hence, it is a kind of effective mechanism which can reduce the need of labeled data. In order to detect the pairwises of lncRNAs and proteins that can interact with each other, the state-of-the-art statistical methods have been exploited, such as Recursive Least Squares (RLS), Kronecker RLS, Sparse Representation based Classifier (SRC), and Multiple Kernel Learning (MKL). All these techniques have already been applied in predicting Protein-Protein Interactions (PPIs) (Ding et al., 2016; Liu X. et al., 2016), Drug-Target Interactions (DTIs) (Xia Z. et al., 2010; Laarhoven et al., 2011; Twan and Elena, 2013; Nascimento et al., 2016; Shen et al., 2017b), binding sites of biomolecules (Ding et al., 2017; Shen et al., 2017a) identification of disease-resistant genes (Xia J. et al., 2010), and microRNA-disease associations (Zou et al., 2015; Peng et al., 2017) with comparative consequences.

With reference to the above researches, we have enriched the categories of similarity measures adopted during LPI prediction. Integration of the heterogeneous kinds of similarity information is achieved by applying Fast Kernel Learning (FastKL) which deals with kernel weight optimization. This is done through the integration of the prediction architectures for weighting heterogeneous kernels. This research proposes a kind of two-step Kernel Ridge Regression (KRR) applied in the field of LPI prediction. LPI-FKLKRR has proven to be a more reliable and effective approach for LPI prediction, compared with other competitive methods. The core of the algorithm proposed herein has been evaluated on the benchmark dataset of LPIs. What is especially encouraging, is that many of the LPI predictions made by our method have been confirmed, with a high degree of correlation. Also, we have conducted a comparative testing on a novel dataset to illustrate the stable performance of the LPI-FKLKRR.

## 2. Methods

In this section, we focus on the elaboration of architecture for our model. Its basic structural components-entities are the following: The known interactions matrix of LPI and the multivariate information that consists of lncRNA expressions, the local network, the sequence information and moreover the Gene Ontology (GO). It is imperative to combine all the similarity information together with the respective combination weights. Finally, we have developed and employed the *LPI with Fast Kernel Learning based on Kernel Ridge Regression Prediction* (LPI-FKLKRR) identification strategy, which utilizes a kind of two-stage Kernel Ridge Regression in LPI prediction.

### 2.1. Problem Specification

Suppose there are *m* lncRNAs and *n* proteins involved in LPI. We formally define two kinds of molecules as $L=\left\{l_{i}\;|i=1,2,\cdots \;,m\right\}$ and $P=\left\{p_{j}\;|j=1,2,\cdots \;,n\right\}$, respectively. Hence, the interactions between lncRNAs and proteins can be intuitively and succinctly expressed as an adjacency matrix **F** with *m* × *n*, which can be formulated as Equation (1)

(1) $$F={\left[\begin{array}{cccccc} f_{1,1} & f_{1,2} & \cdots & f_{1,j} & \cdots & f_{1,n}\\ f_{2,1} & f_{2,2} & \cdots & f_{2,j} & \cdots & f_{2,n}\\ \vdots & \vdots & \ddots & \vdots & \ddots & \vdots \\ f_{i,1} & f_{i,2} & \cdots & f_{i,j} & \cdots & f_{i,n}\\ \vdots & \vdots & \ddots & \vdots & \ddots & \vdots \\ f_{m,1} & f_{m,2} & \cdots & f_{m,j} & \cdots & f_{m,n} \end{array}\right]}_{m\times n}$$

where *f*_*i, j*_ in matrix **F** corresponds to the prediction value of pairwise 〈*l*_*i*_, *p*_*j*_〉, 1 ≤ *i* ≤ *m*, 1 ≤ *j* ≤ *n*, and *m, n* ∈ ℕ^*^. If lncRNA *l*_*i*_ can interact with protein *p*_*j*_, the value of *f*_*i, j*_ is marked as 1, otherwise it is marked as 0.

Obviously, the identification of new interactions between lncRNAs and proteins can be viewed as a task suitable for a recommender system (Koren et al., 2009) of a bipartite network, which can mine and detect the potential associated individuals. To this end, we use Multiple Kernel Learning (MKL) to design the optimization with respect to the prediction of LPI. In the following chapter, we will support the argument that the similarity matrix is equivalent to a kernel.

### 2.2. lncRNA Kernels and Protein Kernels

**Figure 1:**
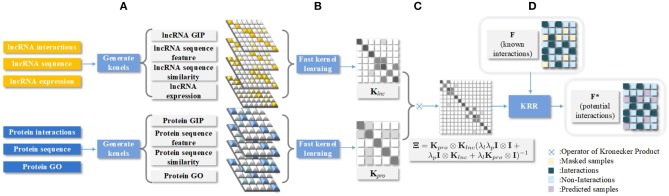
Technical flow chart of our LPI prediction model. **(A)** LncRNAs and proteins belong to two separated and independent spaces, respectively; **(B)** Fast kernel learning is applied to estimate the weight of each kernel in the corresponding space; **(C)** Kronecker Product is adopted in generating the final kernel matrix; **(D)** Kernel Ridge Regression (KRR) is applied in LPI prediction.

In order to conduct MKL, it is inevitable to construct similarity matrices of molecules in lncRNA and protein kernel spaces, respectively. Specifically, lncRNA expression, protein GO, lncRNA sequence, protein sequence, and known interactions between one lncRNA and all proteins are considered in our framework. In addition, the training adjacency matrix **F**_*train*_ is obtained by masking the known pairwise 〈*l*_*i*_, *p*_*j*_〉, where the partial known elements in the matrix are set to 0 for the validation set, which are represented in Figure 1.

#### 2.2.1. Gaussian Interaction Profile Kernel

Interactions can be reflected in the connectivity behavior in the subjacent network (Laarhoven et al., 2011; Twan and Elena, 2013). For the lncRNAs, we extract information of lncRNA interactions corresponding to each row of the training adjacency matrix **F**_*train*_. Then we use a broadly applicable Gaussian Interaction Profile (GIP) kernel to device interaction kernel defined for lncRNA *l*_*i*_ and *l*_*k*_ (*i, k* = 1, 2, ⋯ , *m*). GIP about protein *p*_*j*_ and *p*_*s*_ (*j, s* = 1, 2, ⋯ , *n*) can be generated in a similar way. As a summary, each element value in GIP can be represented as follows:

(2a) $$K_{GIP}^{lnc}(l_{i},l_{k})=exp(-\sigma_{lnc}\|F_{l_{i}}-F_{l_{k}}{\|}^{2})$$

(2b) $$K_{GIP}^{pro}(p_{j},p_{s})=exp(-\sigma_{pro}\|F_{p_{j}}-F_{p_{s}}{\|}^{2})$$

where **F**_*l*_*i*__, **F**_*l*_*k*__ and **F**_*p*_*j*__, **F**_*p*_*s*__ are the matrices of interactions for lncRNA *l*_*i*_, *l*_*k*_ and protein *p*_*j*_, *p*_*s*_, respectively. The Gaussian kernel bandwidths σ_*lnc*_ and σ_*pro*_ are initialized to the value of 1 in the experiments. Practically, when employing 5-fold CV and LOOCV, the GIP kernel similarity should be recalculated each time based on the training samples.

#### 2.2.2. Sequence Similarity Kernel

A sequence *S* with length *d* is an ordered list of characters, which can be written as *S* = *c*_1_*c*_2_⋯  *c*_*h*_⋯  *c*_*d*_ (1 ≤ *h* ≤ *d*). Enlightened by state-of-the-art methods (Yamanishi et al., 2008; Nascimento et al., 2016), we use normalized Smith-Waterman (SW) score (Smith and Waterman, 1981) to measure the sequence similarity. The formulations are represented as follows:

(3a) $$K_{SW}^{lnc}(l_{i},l_{k})=SW(S_{l_{i}},S_{l_{k}})/\sqrt{SW(S_{l_{i}},S_{l_{i}})SW(S_{l_{k}},S_{l_{k}})}$$

(3b) $$K_{SW}^{pro}(p_{j},p_{s})=SW(S_{p_{j}},S_{p_{s}})/\sqrt{SW(S_{p_{j}},S_{p_{j}})SW(S_{p_{s}},S_{p_{s}})}$$

where *SW*(·, ·) stands for Smith-Waterman score; *S*_*l*_*i*__ and *S*_*l*_*k*__ are the sequences for lncRNA *l*_*i*_ and *l*_*k*_; *S*_*p*_*j*__ and *S*_*p*_*s*__ denote the sequences for protein *p*_*j*_ and *p*_*s*_.

#### 2.2.3. Sequence Feature Kernel

We obtain the sequence feature kernel by extracting the feature of the sequences about lncRNAs and proteins. In practice, Conjoint Triad (CT) (Shen et al., 2007) and Pseudo Position-Specific Score Matrix (Pse-PSSM) (Chou and Shen, 2007) are adopted to describe lncRNA and protein sequences, respectively. Both Sequence Feature kernels (SF) ${\text{K}}_{SF}^{lnc}$ and ${\text{K}}_{SF}^{pro}$ are constructed based on a Radial Basis Function kernel (RBF) with bandwidth equals to 1.

#### 2.2.4. lncRNA Expression Kernel

It is interesting to identify genes with concordant behaviors because different genes always show different behaviors (Lai et al., 2017). Expression profiles of lncRNAs refers to 24 cell types which come from NONCODE database (Xie et al., 2014). After expressing each lncRNA as a 24-dimensional expression profile vector, the kernel of lncRNAs expression ${\text{K}}_{EXP}^{lnc}$ can be generated according to the RBF, and kernel bandwidth is also set to 1.

#### 2.2.5. GO Kernel

Inspired by a former research (Zheng et al., 2012), similar Gene Ontology (GO) with proteins are expected to act in similar biological processes, or to reside in similar cell compartments, or to have similar molecular functions. Therefore, GO annotations are employed in this paper to generate a similarity matrix in protein space. The files of Gene Ontology (GO) terms have been downloaded from the GOA database (Wan et al., 2013).

Semantic similarity is always based on the overlap of the terms associated with two proteins (Wu et al., 2013). Jaccard value which we exploited in measuring the semantic similarity of two GO terms *t*_*j*_ and *t*_*s*_ related to proteins *p*_*j*_ and *p*_*s*_ is defined as follows:

(4) $$Jaccard(t_{j},t_{s})=\frac{\left|t_{j}\cap t_{s}\right|}{\left|t_{j}\cup t_{s}\right|}$$

where *t*_*j*_ ∩ *t*_*s*_ denotes the common terms between *p*_*j*_ and *p*_*s*_, and *t*_*j*_ ∪ *t*_*s*_ refers to total number of terms of *p*_*j*_ and *p*_*s*_. However, there has not been any formal definition with GO common terms *t*_*j*_∩*t*_*s*_ given before.

We denote that, if the two sequences are completely consistent, two sequences *S*_1_ and *S*_2_ have common terms of GO. For example, given three sequences *S*_1_ = 〈3, 1, 5〉, *S*_2_ = 〈3, 2, 5〉, and *S*_3_ = 〈3, 2, 5〉, if we only follow that all the corresponding locations of three sequences have non-zero values, then all three sequences have common terms. Nevertheless, for sequence *S*_2_, it can be said that *S*_2_ has common terms with *S*_3_, but does not have common terms with *S*_1_, because the second characters of *S*_1_ and *S*_2_ are different. Thus, we obtain a more sparse GO similarity matrix ${\text{K}}_{GO}^{pro}$ which can facilitate the computation.

### 2.3. Fast Kernel Learning

In MKL, we need to find an optimal mapping vector **w**, i.e., we require to choose a kind of optimal weighting strategy so that object similarity matrices can be appropriately constructed. Concretely, the vector of parameter weight values for lncRNA kernels and protein kernels are represented as **w**^*lnc*^ and **w**^*pro*^, respectively. We have already described that there are four kernels in lncRNA space including ${\text{K}}_{GIP}^{lnc}$, ${\text{K}}_{SW}^{lnc}$, ${\text{K}}_{SF}^{lnc}$, and ${\text{K}}_{EXP}^{lnc}$, and four kernels in protein space including ${\text{K}}_{GIP}^{pro}$, ${\text{K}}_{SW}^{pro}$, ${\text{K}}_{SF}^{pro}$, and ${\text{K}}_{GO}^{pro}$, respectively. The optimal lncRNA and protein kernels are given as follows:

(5a) $$K_{lnc}=\sum_{a=1}^{4}w_{a}^{lnc}K_{a}^{lnc},\;K_{a}^{lnc}\in {ℜ}^{m\times m}$$

(5b) $$K_{pro}=\sum_{a=1}^{4}w_{a}^{pro}K_{a}^{pro},\;K_{a}^{pro}\in {ℜ}^{n\times n}$$

where $w_{a}^{lnc}$ and $w_{a}^{pro}$ denote each element in **w**^*lnc*^ and **w**^*pro*^; ${\text{K}}_{a}^{lnc}$ and ${\text{K}}_{a}^{pro}$ correspond each kind of normalized similarity matrix among the heterogenous similarity kernels in lncRNA and protein spaces.

According to the description of Fast Kernel Learning (FastKL) (He et al., 2008), **w** is used as a substitute for the required optimal solution **w^lnc^** or **w^pro^**, and **K** denotes kernel matrix **K**_*lnc*_ or **K**_*pro*_. FastKL is not only minimizing the distance between **K** and **Y**, where **Y** = **yy**^T^, **y** is a matrix corresponds to all training set labels. It considers the regularization term ||**w**||^2^ that is used to prevent overfitting. To this end, **w** can be drawn from the Formula 6 as follows:

(6) $$\begin{array}{l} \underset{w,K}{\min}\;\|K-Y{\|}_{F}^{2}+\lambda \|w{\|}^{2}\\ s.t.\;\sum_{a}^{J}w_{a}=1 \end{array}$$

where *F* represents Frobenius norm and λ is the tradeoff parameter. In practice, we set λ 10000 when selecting the optimal parameter value.

As a step forward to deduct Equation (6), since the Frobenius norm of a matrix equals to the trace about the product between the matrix itself and matrix of its transformation, i.e., $||\text{X}|{|}_{F}^{2}=tr\left(\text{X}{\text{X}}^{\text{T}}\right)$, the object function with respect to the optimal solution **w** can be simplified as follows:

$$\underset{w}{\min}\;w^{\text{T}}(A+\lambda I)w-2b^{\text{T}}w$$

(7) $$\begin{array}{l} s.t.\;\sum_{a}^{J}w_{a}=1\\ \;A_{u,v}=tr(K_{u}^{\text{T}}K_{v})\\ \;b_{v}=tr(Y^{\text{T}}K_{v}) \end{array}$$

where tr(·) is the symbol of the trace operator; *A*_*u, v*_ represents each element in matrix **A**; **K**_*u*_ and **K**_*v*_ denote two different kernel matrices.

Recapitulating the above statement, through gaining the final **w^lnc^** and **w^pro^**, we have achieved the goal of MKL for fusing all kinds of similarity matrices so that the input matrix of KRR can be generated.

### 2.4. Kernel Ridge Regression

**Figure 2:**
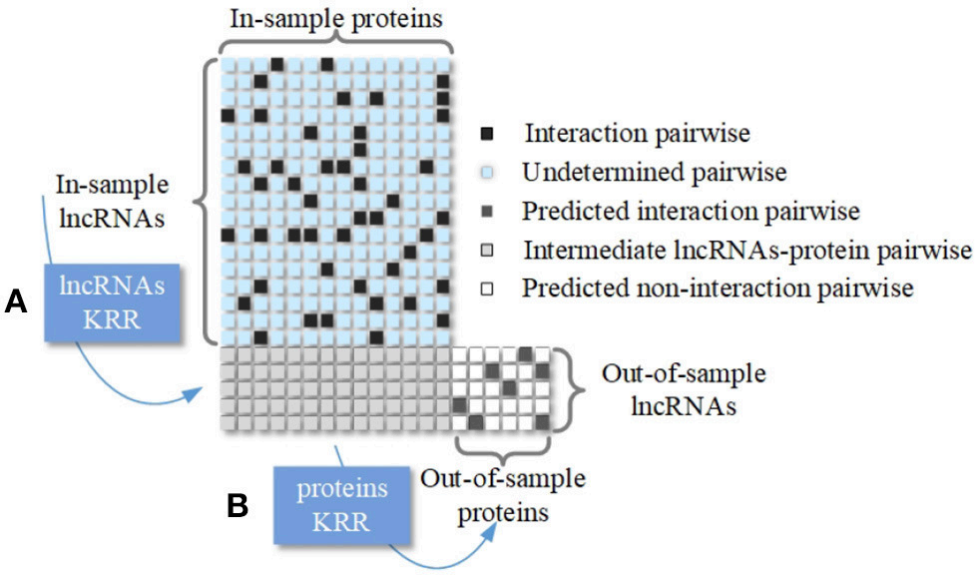
Schematic diagram of two-step Kernel Ridge Regression. **(A)** An intermediate prediction of LPI is conducted using an lncRNA KRR model. **(B)** Protein KRR is trained using the last step information for predicting new proteins.

Stock et al. developed a scenario of pairwise learning, called Kernel Ridge Regression (KRR) (Stock et al., 2016), which can be applied in binary classification. The basic idea of KRR is to minimize a suitable objective function with an *L*_2_-complexity penalty so that it can fit the labeled dyads as much as possible. Specifically, the KRR prediction for the LPI pairwise 〈*l*_*i*_, *p*_*j*_〉 has two steps which are shown in Figure 2.

In the first step, a prediction with respect to the new protein for all intermediate LPI pairwise is obtained as an 1 × *n* vector **f**_*i*, ·_, which can be computed as follows:

(8) $$f_{i,\cdot }=k_{lnc}^{\text{T}}{\left(K_{lnc}+\lambda_{l}I\right)}^{-1}F$$

where **k**_*lnc*_ denotes the vector of lncRNA kernel evaluation between lncRNAs in the training set and a protein in the test set, and λ_*l*_ is the regularization parameter.

In the second step, we can obtain each element $f_{i,j}^{*}$ in the prediction matrix F^*^ by using another regularization parameter λ_*p*_ as following Equation (9):

(9) $$f_{i,j}^{*}=k_{pro}^{T}{\left(K_{pro}+\lambda_{p}I\right)}^{-1}f_{i,\cdot }^{T}$$

Considering the optimal lncRNAs and proteins kernels **K**_*lnc*_ and **K**_*pro*_, the general objective function of the two-step KRR is defined as follows:

(10) $$\underset{F^{*}}{\min}\sum_{(i,j,f)\in F}{\left(f_{i,j}-f_{i,j}^{*}\right)}^{2}+vec{\left(F^{*}\right)}^{\text{T}}\Xi^{-1}vec\left(F^{*}\right)$$

where *vec*(·) is a vectorization operator that can rearrange the matrix elements in one row; F^*^ denotes the prediction of the original matrix **F** which can be estimated with the application of the LPI-KRR. Objective function in Equation (8) need to be minimized by iterations, and the iterations usually gets converged in about 5–10 iterations.

The kernel matrix **Ξ** that is used in Equation (10) is defined as Equation (11):

(11) $$\Xi =K_{pro}⊗K_{lnc}{\left(\lambda_{l}\lambda_{p}I⊗I+\lambda_{p}I⊗K_{lnc}+\lambda_{l}K_{pro}⊗I\right)}^{-1}$$

By using the lncRNAs, the proteins' kernels and the two regularization parameters λ_*l*_ and λ_*p*_, each element in matrix F^*^ can be represented as Equation (12):

(12) $$F^{*}=K_{lnc}{\left(K_{lnc}+\lambda_{l}I\right)}^{-1}F{\left(K_{pro}+\lambda_{p}I\right)}^{-1}K_{pro}$$

The LPI-FKLKRR calculation framework is illustrated in the following Algorithm 1.

**Algorithm 1:** Fast Kernel Learning based on Kernel Ridge Regression (LPI-FKLKRR).

**Input:** ${\text{K}}_{GIP}^{lnc}$, ${\text{K}}_{SW}^{lnc}$, ${\text{K}}_{SF}^{lnc}$, ${\text{K}}_{EXP}^{lnc}$ ∈ℜ^*m*×*m*^ and ${\text{K}}_{GIP}^{pro}$, ${\text{K}}_{SW}^{pro}$, ${\text{K}}_{SF}^{pro}$, ${\text{K}}_{GO}^{pro}$ ∈ℜ^*n*×*n*^; **F**∈ℜ^*m*×*n*^.
Output: F^*^.
1: Calculate **w^lnc^** and **w^pro^** and adjust the parameter λ by Eq.??;
2: Calculate **K**_*lnc*_ and **K**_*pro*_ by using Eq.4a and Eq.4b;
3: Calculate the prediction value in matrix F^*^ by Eq.10;
4: Adjust the parameters λ_*l*_ and λ_*p*_ by using Eq.8 and Eq.9, and produce the optimal F^*^.

## 3. Results

This section provides a quantitative evaluation that employ benchmark dataset to assess our approach. We first show a result of 5-fold cross validation, then conduct an independent analyzing about performance of single kernel. Moreover, LPI-FKLKRR is not only compared with mean weighted model but also be assessed in parallel comparison including other outstanding methods. Furthermore, we utilize the case study to evaluate our method in predicting unknown lncRNA-protein interactions. What's more, there is also a comparison between LPI-FKLKRR and state-of-the-art work on a novel dataset.

### 3.1. Benchmark Dataset

Although there exists a high volume of web-based resources (Park et al., 2014), available datasets should be carefully selected. We have acquired the benchmark dataset according to the state-of-the-art work by Zhang et al. (2017). They have experimentally determined lncRNA-protein interactions with 1114 lncRNAs and 96 proteins from NPInter V2.0 (Yuan et al., 2014). Non-coding RNAs and sequence information of proteins were gleaned from NONCODE (Xie et al., 2014) and SUPERFAMILY database (Gough et al., 2001), respectively. Zhang et al. also removed lncRNAs and proteins whose expression or sequence information were unavailable in order to reduce the pressure of computation. Those lncRNAs and proteins with only one interaction were removed for the same reason. A dataset with 4158 lncRNA-protein interactions which contains 990 lncRNAs and 27 proteins were finally collected.

### 3.2. Evaluation Measurements

To gauge the stability of our model, 5-fold Cross Validation (5-fold CV) has been employed. The Area Under ROC curve (AUC) and Area Under the Precision-Recall curve (AUPR) measures have been utilized to evaluate our approach. We would like to emphasize that AUPR is more significant than AUC as a quality measurement because of the sparsity of the true lncRNA-protein interactions.

### 3.3. Experimental Environment

The proposed LPI-FKLKRR algorithm, has been implemented by using MATLAB as the development and compilation platform. All programs have been validated on a computer with 3.7 GHz 4-core CPU, 20 GB of memory, and 64-bit Windows Operating Systems.

### 3.4. Parameter Optimization

Grid search schema has been adopted to get the optimized values of the parameters λ_*l*_ and λ_*p*_. The range of λ_*l*_ is from 20 to 980 while λ_*p*_ parameter ranges from 2 to 27. The criteria used to select the optimal values of λ_*l*_ and λ_*p*_ were the highest AUPR value and the lowest values of λ_*l*_ and λ_*p*_, due to the fact that the smaller values of λ_*l*_ and λ_*p*_, the less is the running time of the algorithm. We have found that λ_*l*_ = 20.89 and λ_*p*_ = 0.02 are the best values for the two parameters (AUPR: 0.6950).

### 3.5. Performance Analysis

**Figure 3:**
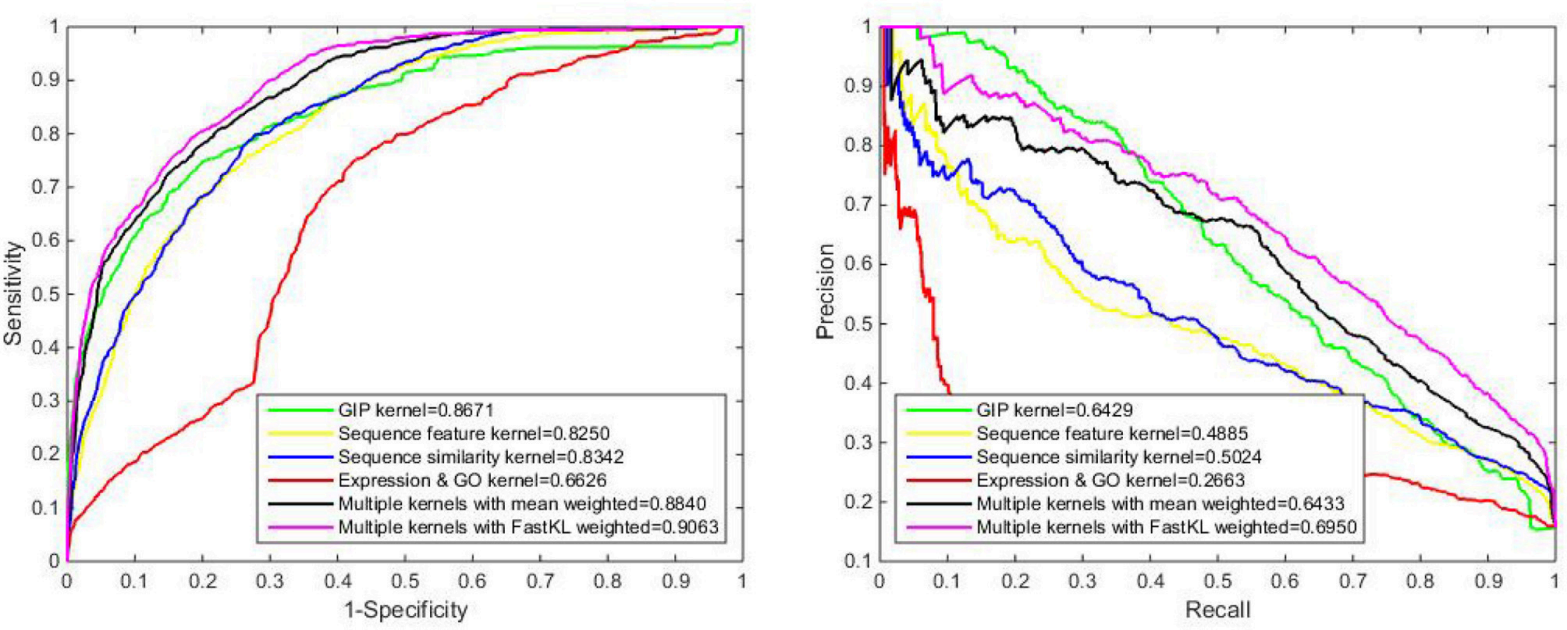
The ROC and PR curve of different models.

After testing different kinds of kernels on the benchmark dataset, we obtain that the AUPRs of GIP kernel, sequence feature kernel, sequence similarity kernel and gene expression & protein GO kernel are 0.6429, 0.4885, 0.5024, and 0.2663, respectively. The detailed results are listed in Table 1. It is obvious that GIP kernel has the highest AUPR value (among the single Kernels). Multiple kernels with the FastKL weighted model achieves AUPR equal to 0.6950, which is an outstanding performance. In Figure 3, we can see that the FastKL performs better than the other models. It is clear that the FastKL is effective in improving the performance of LPIs prediction.

**Table 1:** The AUPR and AUC of different kernels on benchmark dataset.

**Kernel type**	**AUPR**	**AUC**
GIP kernel	0.6429	0.8671
Sequence feature kernel	0.4885	0.8250
Sequence similarity kernel	0.5024	0.8342
Gene expression & protein GO	0.2663	0.6626
Multiple kernels with mean weighted	0.6433	0.8840
Multiple kernels with FastKL weighted	**0.6950**	**0.9063**

*Bold values represent the best value in columns*.

**Figure 4:**
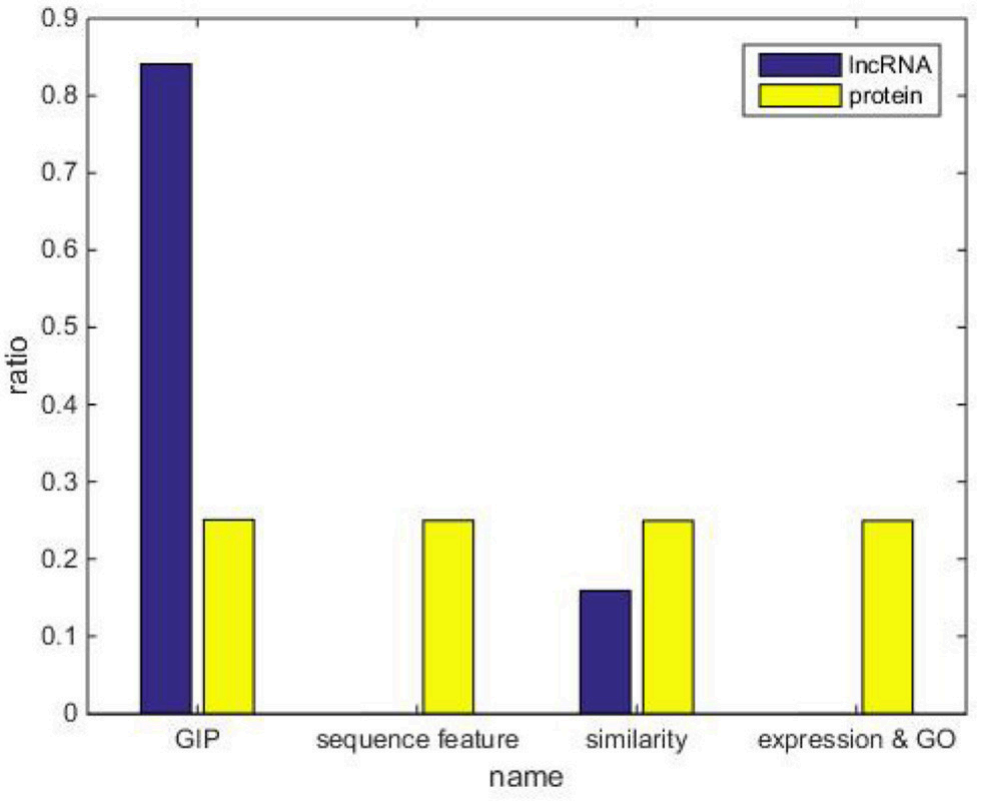
The kernel weights in the experiment of LPI-FKLKRR on benchmark dataset.

In addition, Figure 4 shows the weight of each kernel, including lncRNA space and protein space in a 5-fold CV experiment. Conspicuously, weights of GIP kernel obtain the largest values on the lncRNA space. However, four kinds of protein similarity matrices equally divide the weights in protein space. This occasion should be explained that four kinds of protein similarity have low degree of overlapping in the representation space, i.e., each kind of protein similarity presents a specific aspect of protein feature.

### 3.6. Comparing to Existing Predictors

The comparison between our approach and other existing methods are showed in Table 2. It should be mentioned that the highest AUPR 0.6950 is achieved by our proposed approach, which is superior to all others. The AUPR values for the other established methods are the following: integrated LPLNP (AUPR: 0.4584) (Zhang et al., 2017), RWR (AUPR: 0.2827) (Gan, 2014), CF (AUPR: 0.2357) (Sarwar et al., 2001), LPIHN (AUPR: 0.2299) (Li et al., 2015), and LPBNI (AUPR: 0.3302) (Ge et al., 2016). There are two well-founded reasons for the successful improved performance of our method. Firstly, FastKL effectively combines multivariate information by employing multiple kernel learning. Simultaneously, LPI-KRR is an effective prediction algorithm employing two-step KRR to fuse lncRNA and protein feature spaces. Due to the fact that there are extrapolation difficulties for the imbalanced datasets, PRC is more effective than ROC on highly imbalanced datasets. Therefore, we have obtained acquire competitive AUC value, compared to the state-of-the-art algorithms. From all the above we conclude that our approach can be a useful tool in the prediction of LPI.

**Table 2:** Comparison to existing methods via 5-fold CV on benchmark dataset.

**Method**	**AUPR**	**AUC**
LPI-FKLKRR	**0.6950**	0.9063
Integrated LPLNP^*^	0.4584	**0.9104**
RWR^*^	0.2827	0.8134
CF^*^	0.2357	0.7686
LPIHN^*^	0.2299	0.8451
LPBNI^*^	0.3302	0.8569

* *Results are derived from Zhang et al. (2017). Bold values represent the best value in columns*.

### 3.7. Case Study

**Figure 5:**
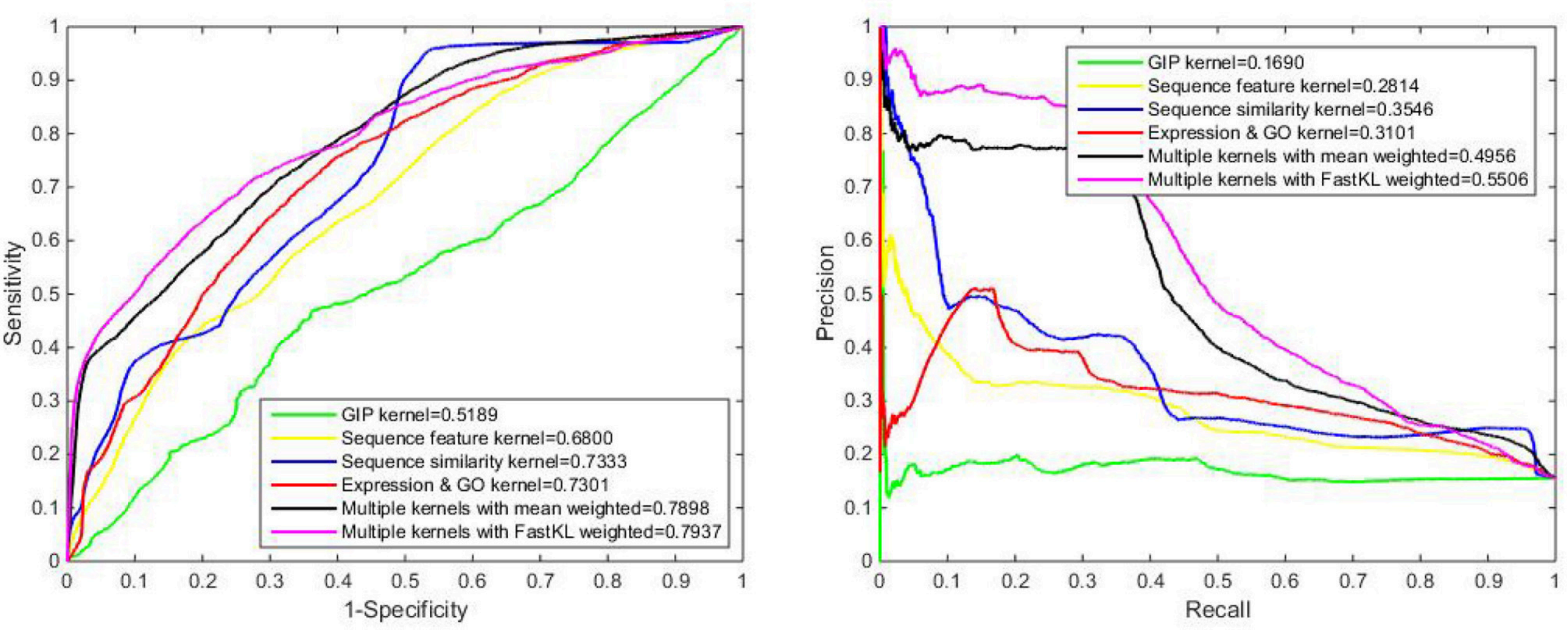
The ROC and PR curve by local LOOCV on benchmark dataset.

We have also used Local Leave-One-Out Cross-Validation (LOOCV) to evaluate the predictive performance. Local LOOCV masks the relationship between one protein and all lncRNAs. Our model is trained by the rest of the known information no matter if they are interacting or not and it is tested on a masked relationship. For a protein not appearing in the trial, our approach can predict the strength of interactions between this protein and gross 990 lncRNAs in the experiment. We have ranked these values of interactions in descending order, since high ranking is connected to high interaction possibility. In Figure 5, we can see that the performance of single kernel, average weighted kernels and weighted kernels with FastKL have failed. The FastKL weighted model using Multiple kernels, gains the best performance with values 0.5506 and 0.7937 for the AUPR and the AUC respectively. The detailed results are listed in Table 3.

**Table 3:** The AUPR and AUC of different kernels by local LOOCV on benchmark dataset.

**Kernel**	**AUPR**	**AUC**
GIP kernel	0.1690	0.5189
Sequence feature kernel	0.2814	0.6800
Sequence similarity kernel	0.3546	0.7333
Gene expression & protein GO	0.3101	0.7301
Multiple kernels with mean weighted	0.4956	0.7898
Multiple kernels with FastKL weighted	**0.5506**	**0.7937**

*Bold values represent the best value in columns*.

As shown in Table 4, two cases of the top 20 interactions (including proteins ENSP00000309558 and ENSP00000401371), have been extrapolated by LPI-FKLKRR. Also, two cases in Table 5 including lncRNAs, NONHSAT145960 and NONHSAT031708 of the top 10 interactions have been extrapolated by the LPI-FKLKRR. We check them up in the masked relationship between one protein and all lncRNAs, or one lncRNA and all proteins. Our approach achieves successful identification proportion equal to 11/20 and 12/20 on the proteins ENSP00000309558 and ENSP00000401371, respectively, and it achieves identification proportion equal to 6/10 and 6/10 on lncRNAs NONHSAT145960 and NONHSAT031708.

**Table 4:** Top 20 interactions rank on protein ENSP00000309558 and ENSP00000401371.

**lncRNA ID**	**Protein ID**	**Rank**	**Confirm?**	**lncRNA ID**	**Protein ID**	**Rank**	**Confirm?**
NONHSAT011652	ENSP00000309558	1	Confirmed	NONHSAT002344	ENSP00000401371	1	Confirmed
NONHSAT027070	ENSP00000309558	2	Confirmed	NONHSAT104639	ENSP00000401371	2	–
NONHSAT104991	ENSP00000309558	3	Confirmed	NONHSAT027070	ENSP00000401371	3	Confirmed
NONHSAT001511	ENSP00000309558	4	Confirmed	NONHSAT104991	ENSP00000401371	4	Confirmed
NONHSAT079374	ENSP00000309558	5	–	NONHSAT101154	ENSP00000401371	5	–
NONHSAT009703	ENSP00000309558	6	Confirmed	NONHSAT041921	ENSP00000401371	6	Confirmed
NONHSAT138142	ENSP00000309558	7	Confirmed	NONHSAT042032	ENSP00000401371	7	–
NONHSAT104639	ENSP00000309558	8	Confirmed	NONHSAT131038	ENSP00000401371	8	Confirmed
NONHSAT135796	ENSP00000309558	9	Confirmed	NONHSAT084827	ENSP00000401371	9	–
NONHSAT077129	ENSP00000309558	10	–	NONHSAT021830	ENSP00000401371	10	Confirmed
NONHSAT023404	ENSP00000309558	11	–	NONHSAT001953	ENSP00000401371	11	Confirmed
NONHSAT063901	ENSP00000309558	12	Confirmed	NONHSAT145923	ENSP00000401371	12	Confirmed
NONHSAT099046	ENSP00000309558	13	–	NONHSAT039675	ENSP00000401371	13	–
NONHSAT031489	ENSP00000309558	14	–	NONHSAT135796	ENSP00000401371	14	Confirmed
NONHSAT041921	ENSP00000309558	15	Confirmed	NONHSAT011652	ENSP00000401371	15	Confirmed
NONHSAT013639	ENSP00000309558	16	–	NONHSAT044002	ENSP00000401371	16	–
NONHSAT027206	ENSP00000309558	17	–	NONHSAT112849	ENSP00000401371	17	–
NONHSAT134595	ENSP00000309558	18	–	NONHSAT114444	ENSP00000401371	18	Confirmed
NONHSAT054716	ENSP00000309558	19	–	NONHSAT007429	ENSP00000401371	19	Confirmed
NONHSAT122291	ENSP00000309558	20	Confirmed	NONHSAT123220	ENSP00000401371	20	–

**Table 5:** Top 10 interactions rank on lncRNA NONHSAT145960 and NONHSAT031708.

**lncRNA ID**	**Protein ID**	**Rank**	**Confirm?**	**lncRNA ID**	**Protein ID**	**Rank**	**Confirm?**
NONHSAT145960	ENSP00000258962	1	–	NONHSAT031708	ENSP00000385269	1	Confirmed
NONHSAT145960	ENSP00000240185	2	Confirmed	NONHSAT031708	ENSP00000258962	2	–
NONHSAT145960	ENSP00000385269	3	–	NONHSAT031708	ENSP00000240185	3	Confirmed
NONHSAT145960	ENSP00000349428	4	Confirmed	NONHSAT031708	ENSP00000349428	4	–
NONHSAT145960	ENSP00000379144	5	Confirmed	NONHSAT031708	ENSP00000258729	5	Confirmed
NONHSAT145960	ENSP00000338371	6	Confirmed	NONHSAT031708	ENSP00000338371	6	–
NONHSAT145960	ENSP00000401371	7	Confirmed	NONHSAT031708	ENSP00000379144	7	–
NONHSAT145960	ENSP00000254108	8	–	NONHSAT031708	ENSP00000254108	8	Confirmed
NONHSAT145960	ENSP00000258729	9	Confirmed	NONHSAT031708	ENSP00000401371	9	Confirmed
NONHSAT145960	ENSP00000413035	10	–	NONHSAT031708	ENSP00000371634	10	Confirmed

### 3.8. Speed Comparison on Benchmark Dataset

Practically, running speed is also play an important role in predicting LPI. The state-of-the-art methods of peer groups, such as LPLNP, can produce high-accuracy performances. Hence, the overall evaluation of the success of each approach, should also consider the Running Time (RT). Thus, a comparison between the RT of LPLNP and LPI-FKLKRR, has been performed. The comparative RT analysis between LPLNP and LPI-FKLKRR after running the available source code of LPLNP from the network, is illustrated in Table 6.

**Table 6:** Comparison of running time between LPI-FKLKRR and LPLNP in 10 times.

**Method**	**Average running time(s)**	**Standard deviation(s)**
LPI-FKLKRR	**11.48**	**0.2126**
LPLNP^a^	352.93	2.6656

a *The address of LPLNP is given by Zhang et al. (2017). Bold values represent the best value in columns*.

Although LPLNP and LPI-FKLKRR have competitive AUC (according to results shown in Table 2) it is clear that the LPI-FKLKRR achieves better average running performance using only 11.48 s to accomplish the prediction task of LPI. This is much faster than the 352.93 s of the LPLNP (as shown in Table 6. Moreover, the standard deviation also manifest that LPI-FKLKRR is both fast and stable. Furthermore, considering the higher AUPR value of LPI-FKLKRR, we can strongly suggest that LPI-FKLKRR can be both a time-saving and useful tool for LPI prediction.

### 3.9. Evaluation on Novel Dataset

To support the results of the benchmark experiments, we have employed another dataset which is published by Zheng et al. The size of the novel dataset is larger than the benchmark dataset, which is shown in Table 7.

**Table 7:** The information of two datasets in the experiment.

**Dataset**	**Number of lncRNAs**	**Number of proteins**	**LPIs**
benchmark dataset^*^	990	27	4,158
novel dataset^*^	1,050	84	4,467

* *The benchmark dataset and the novel dataset come from the paper of Zhang et al. (2017) and Zheng et al. (2017), respectively*.

Originated from the same databases as the benchmark dataset, the novel dataset consists of 4467 LPIs, including 1050 unique lncRNAs and 84 unique proteins. We have conducted the comparison of LPI-FKLKRR and PPSNs (Zheng et al., 2017) by applying 5-fold CV on novel dataset, and list the results in Table 8. The AUC value for the LPI-FKLKRR algorithm is equal to 0.9669, which is higher than the one of PPSNs. What's more, the AUPR value which is equal to 0.7062 for the novel dataset proves the robustness performance of the LPI-FKLKRR on an imbalanced dataset.

**Table 8:** The AUPR and AUC of different methods on novel dataset.

**Method**	**AUPR**	**AUC**
LPI-FKLKRR	**0.7062**	**0.9669**
PPSNs	–^a^	0.9098
NRLMF	0.4010	0.8287
CF	0.4267	0.8103

a *AUPR is not exploited by Zheng et al. (2017). Bold values represent the best value in columns*.

Apart from the baseline methods that we have done test in Figure 2, we make a new comparison on the dataset that proposed by Zheng et al. with methods including NRLMF and CF. NRLMF, which is also capable of integrating various data sources, achieved good performance for both MDA prediction (Yan et al., 2017; He et al., 2018) and DTI prediction (Liu Y. et al., 2016). And CF method that has proposed by Sarwar et al., is another state-of-the-art work. From Table 8, we notice that no matter from the aspect of AUPR or AUC, the value of LPI-FKLKRR are higher than NRLMF (AUPR:0.4010, AUC:0.8287) and CF (AUPR:0.4267, AUC:0.8103).

**Figure 6:**
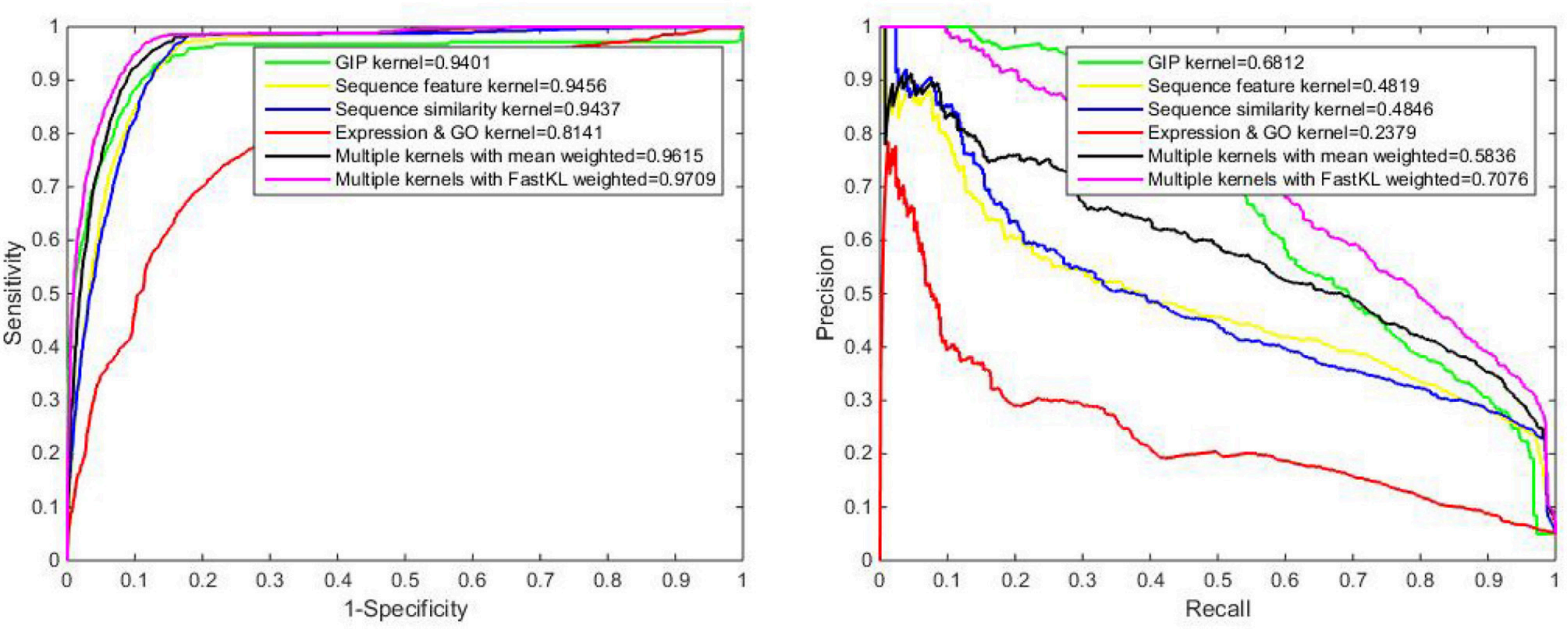
The ROC and PR curve of different models with novel dataset by 5-fold CV.

**Figure 7:**
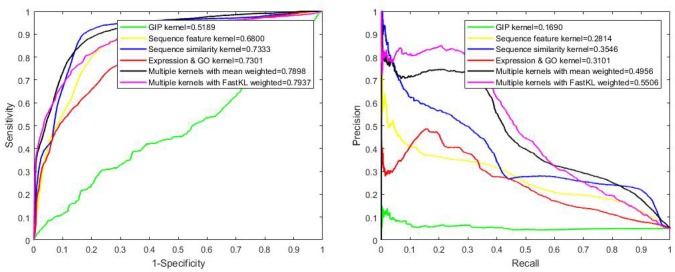
The ROC and PR curve by local LOOCV on novel dataset.

Both the 5-fold CV and local LOOCV are also done in the novel dataset experiment. After testing different kinds of kernels on the novel dataset, we obtain that in the 5-fold CV, the AUPRs of GIP kernel, sequence feature kernel, sequence similarity kernel and gene expression & protein GO kernel are 0.6812, 0.4819, 0.4846, and 0.2379, respectively. Multiple kernels with the FastKL weighted model achieves AUPR equal to 0.7076, which is an outstanding performance. In Figures 6, 7, we can see that the FastKL performs better than the other models. This result is consistent with the consequence on benchmark dataset.

## Conclusions and Discussion

In this paper, we have proposed a novel prediction method for the prediction of lncRNAs-protein interactions by using Kernel Ridge Regression, combined with a multiple kernel learning approach (LPI-FKLKRR). LPI-FKLKRR employs fast kernel learning to fuse lncRNA and protein similarity matrices, respectively. A two-step Kernel Ridge Regression is adopted to forecast the interactions between lncRNAs and proteins. The 5-fold cross validation (5-fold CV) testing of the proposed LPI-FKLKRR algorithm, achieved very reliable and promising results when applied on the benchmark dataset (AUPR: 0.6950). Furthermore, LPI-FKLKRR achieves satisfactory prediction performances compared with the state-of-the-art approaches. A comparison on a novel dataset illustrates the stability performance of our model.

From the view point of the classification method about the prediction, the problem setting of lncRNA-protein interaction prediction can be the same with miRNA-disease interaction prediction and drug-target interaction prediction (Ezzat et al., 2018). For instance, CF method, which has proposed by Sarwar et al, has a recent work named MSCMF, which projects drugs and targets into a common low-rank feature space Zheng et al. (2013). This method can be transfered to the area of LPI prediction. Ezzat et al. have supposed that chemogenomic methods can be categorized into five types, including neighborhood models, bipartite local models, network diffusion models, matrix factorization models, and feature-based classification models. Consequently, in the future we will improve the predicting performance by adding information such as available 3D structure data, by constructing more heterogeneous similarity matrices, by changing weighting strategy or by drawing other effective regression models.

## Data Availability Statement

The datasets and codes for this study can be found in website https://github.com/6gbluewind/LPI_FKLKRR.

## Author Contributions

FG, YD, and CS conceived and designed the experiments. CS and YD performed the experiments and analyzed the data. FG and CS wrote the paper. FG and JT supervised the experiments and reviewed the manuscript.

### Conflict of Interest Statement

The authors declare that the research was conducted in the absence of any commercial or financial relationships that could be construed as a potential conflict of interest.
